# Impact of preoperative anemia on outcomes in patients undergoing curative resection for gastric cancer: a single‐institution retrospective analysis of 2163 Chinese patients

**DOI:** 10.1002/cam4.1309

**Published:** 2018-01-17

**Authors:** Xuechao Liu, Haibo Qiu, Yuying Huang, Dazhi Xu, Wei Li, Yuanfang Li, Yingbo Chen, Zhiwei Zhou, Xiaowei Sun

**Affiliations:** ^1^ Sun Yat‐sen University Cancer Center State Key Laboratory of Oncology in South China Collaborative Innovation Center for Cancer Medicine Guangzhou 510060 China; ^2^ Department of Gastric Surgery Sun Yat‐sen University Cancer Center Guangzhou China

**Keywords:** Gastric cancer, perioperative transfusions, postoperative complications, preoperative anemia, prognosis

## Abstract

We sought to evaluate whether preoperative anemia was an important determinant of survival in gastric cancer (GC). A single institution cohort of 2163 GC patients who underwent curative resection were retrospectively analyzed. Anemia was defined as a preoperative hemoglobin level <120 g/L in males and <110 g/L in females. Overall survival (OS) was analyzed using the Kaplan–Meier method, and a multivariate Cox proportional hazards model was performed to identify the independent prognostic factor. Anemic patients had a poorer OS compared with nonanemic patients after resection for tumor–nodes–metastasis (TNM) stage III tumors (5‐year OS rate: 32.2% vs. 45.7%, *P* < 0.001) but not stage I (*P*  =  0.480) or stage II (*P*  =  0.917) tumors. Multivariate analysis revealed that preoperative anemia was an independent prognostic factor in TNM stage III (hazard ratio [HR], 1.771; 95% CI, 1.040–3.015; *P *=* *0.035). In a stage‐stratified analysis, preoperative anemia was still independently associated with OS in TNM stages IIIa through IIIc (*P *<* *0.001, *P *=* *0.075, and *P *=* *0.012, respectively), though the association was only marginal in stage IIIb. Of note, preoperative mild anemia had a similar prognostic value in TNM stage III GC. Furthermore, preoperative anemia was significantly associated with more perioperative transfusions, postoperative complications and several nutritional‐based indices, including the prognostic nutritional index (PNI), preoperative weight loss and performance status (all *P *<* *0.05). Preoperative anemia, even mild anemia, was an important predictor of postoperative survival for TNM stage III GC.

## Introduction

Anemia is the most common hematologic abnormality of most cancers, with prevalence rates varying by cancer type and disease stage. It has been assumed that between 30% and 90% of patients with cancer are anemic at diagnosis 1, 2. Iron metabolism disorders, tumor‐associated bleeding, abnormal catabolism of cancer patients, and nutritional deficiencies all play a role in anemic pathogenesis 3, 4. In addition to affecting the quality of life, pretreatment anemia has been found to be a predictor of poor prognosis in many cancers 5, 6, 7. One meta‐analysis of 200 studies suggested that anemia was associated with reduced survival times in patients with lung carcinoma, cervicouterine carcinoma, prostatic carcinoma, head and neck carcinoma, lymphoma, and multiple myeloma 8.

Gastric cancer (GC) is the second leading cause of cancer death worldwide, with a high incidence of recurrence and metastasis 9, 10, 11. Prevention and individualized treatment are seen as the best choices to reduce GC mortality rates 12, 13, 14. Hence, there have been continuing efforts to identify prognostic factors to select high‐risk patients for tailored treatment. A study from Korea, which included 1688 GC patients who underwent curative resection between 1991 and 1995, showed that preoperative anemia was an independent prognostic factor for tumor–nodes–metastasis (TNM) stage I‐II GC 15. Due to the regional differences as well as advancements in surgery and multidisciplinary treatment in the past decade, the impact of preoperative anemia on outcomes in Chinese patients remains unclear.

Therefore, this study was conducted to further validate the prognostic value of preoperative anemia in a large cohort of Chinese patients who underwent curative resection for GC.

## Methods

### Ethics approval and consent to participate

The research project was approved by the Ethical Committee of Sun Yat‐sen University Cancer Center and was conducted in accordance with the Declaration of Helsinki. The study was retrospective in design, and no consent from patients was needed.

### Study population

We enrolled a total of 2163 consecutive GC patients undergoing curative D2 gastrectomy with R0 resection at the Sun Yat‐sen University Cancer Center between January 2001 and December 2014. Patients who met all the eligibility criteria were included in the study. The eligibility criteria are as follows: (1) a complete set of clinicopathological and follow‐up data, (2) no neoadjuvant chemotherapy or radiotherapy, (3) no other synchronous malignancy, (4) no other nonmalignancy‐associated anemia, and (5) no red blood cell transfusion before the blood sample was taken. Ultimately, 2163 patients were enrolled.

Clinicopathological and outcome data were collected by review of the medical records. Blood samples were taken within 2 weeks before surgery. Unintentional weight loss 6 months prior to surgery was recorded at the time of the initial diagnosis. The prognostic nutritional index (PNI) was calculated based on previous studies 16. The perioperative period was defined as 7 days before and after operation. All transfused patients received leukocyte‐depleted packed red blood cells (PRBC) according to standard transfusion guidelines 17. In principle, PRBC transfusion should not be initiated before the hemoglobin levels decline to 7 g/dL or lower per the guidelines of our center. All patients had histologically confirmed stage I–III gastric adenocarcinoma. Tumors were staged according to the seventh edition of the American Joint Committee on Cancer (AJCC) tumor–nodes–metastasis (TNM) classification 18. By multidisciplinary discussion after surgery, 5‐fluorouracil‐based adjuvant chemotherapy or combined treatment was delivered to the postoperative patients with stage II–III GC and no marked comorbidities that would preclude chemotherapy use 19, 20. However, part of stage II‐III patients gave up chemotherapy because of severe comorbidities, unacceptable side effects, unaffordable medical care or other personal considerations. Furthermore, all of stage I patients also did not receive adjuvant chemotherapy based on the current guidelines.

### Follow‐up

Patients were examined every 3 months during the first 2 years and every 6 months thereafter. Conventional examinations included clinical symptoms, physical examinations, laboratory testing, dynamic abdominal computed tomography (CT), and gastroscopic examination. Overall survival (OS) was defined as the duration from the surgery date to either the date of death or the date of the last follow‐up.

### Definition of anemia

Anemic status was assessed within 2 weeks before surgery. Based on the criteria recommended by the National Cancer Institute and the clinical practice guidelines released by the Chinese Society of Clinical Oncology (CSCO), anemia is defined as a preoperative hemoglobin level <120 g/L in males and <110 g/L in females. Mild anemia is defined as a hemoglobin level that is greater than 90, but less than the normal level 21.

### Statistical analysis

Our research adhered to the Strengthening the Reporting of Observational Studies in Epidemiology (STROBE) statement (S1). Data are presented as the means and 95% confidence intervals. The differences among the groups were analyzed using the Pearson's chi‐squared test for categorical variables. OS curves were calculated according to the Kaplan–Meier method and compared using the log‐rank test. Variables in which two‐tailed *P* value was less than 0.05 in the univariate or unadjusted analysis were considered possibly associated with OS. Subsequent multivariate analysis was further performed to identify independent prognostic factors. The following variables were adjusted in a multivariate COX proportional hazards model: age, tumor size, tumor location, lymphatic vessel infiltration (LVI), PNI, preoperative weight loss, complications, anemia, perioperative transfusion, and TNM stage. A two‐sided *P* < 0.05 was considered statistically significant. All of the above statistical analyses were performed with SPSS 19.0 software (SPSS, IBM Corp, Armonk, NY). Propensity score matching was performed with Stata13.0 (StataCorp LP, College Station, TX). In our study, the 1:1 nearest neighbor matching was chosen for the propensity score.

## Results

**Figure 1:**
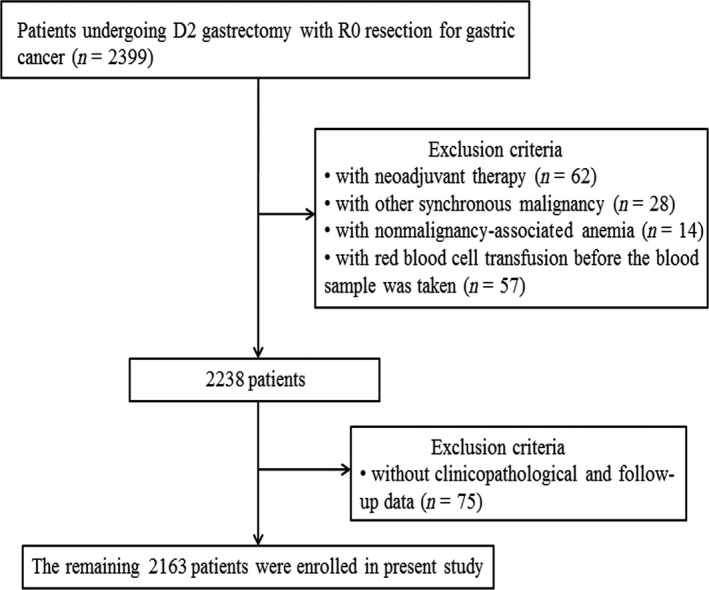
Flowchart of the patient selection.

Among a total of 2163 patients qualified for the analysis, 585 (27.0%) were anemic and 361 (16.7%) were mildly anemic (Fig. 1). The median age at diagnosis was 58 years (range: 14–89 years). Overall, 1442 (66.7%) patients were males and 721 (33.3%) were females (Table 1). There were 451 patients in stage I, 677 in stage II, and 1035 in stage III, and the corresponding number of preoperative anemic patients at each stage was 80 (17.7%), 191 (28.2%), and 314 (30.3%), respectively. Of the 1035 stage III patients, 209 (20.2%) received perioperative PRBC transfusions. The median number of transfused units was 4 (range: 1–24). In addition to the PRBC transfusions, in our study, anemic patients did not receive other preoperative treatment options, including iron therapy and erythropoietic‐stimulating agents. The last follow‐up date was October 30, 2015, and the median follow‐up period was 28 (3–185) months. During the follow‐up period, 655 (30.3%) patients died, and 1508 (69.7%) patients were alive at the end of follow‐up. Among the patients with stage I disease, the 5‐year OS rate was 94.6% in nonanemic patients and 91.8% in anemic patients (*P *=* *0.480; Fig. S1A). In patients with stage II disease, the 5‐year OS rate was 72.9% in nonanemic patients, and 73.2% in anemic patients (*P *=* *0.917; Fig. S1B). For patients with stage III GC, the 5‐year OS rate was 45.7% in nonanemic patients and 32.2% in anemic patients (*P *<* *0.001). The Cox proportional hazards regression models were used to further validate the results in overall patients (HR, 1.238; 95% CI, 0.995–1.539; *P *=* *0.055), stage I patients (HR, 1.007; 95% CI, 0.232–4.383; *P *=* *0.992), and stage II patients (HR, 0.853; 95% CI, 0.453–1.606; *P *=* *0.623), respectively.

**Table 1:** General characteristics of 2163 gastric cancer patients

	No. of patients (%)
Age (years)	
<60	1194 (55.2)
≥60	969 (44.8)
Sex	
Female	721 (33.3)
Male	1442 (66.7)
Tumor size (cm)	
<5	1191 (55.1)
≥5	972 (44.9)
Tumor location	
Upper third	700 (32.4)
Middle third	587 (27.1)
Lower third	876 (40.5)
Grade	
G1/G2	406 (18.8)
G3/G4	1757 (81.2)
Lauren histotype	
Intestinal	431 (29.8)
Diffuse/Mixed	1013 (70.2)
LVI	
Absent	1317 (91.6)
Present	121 (8.4)
PNI	
0	1943 (89.8)
1	220 (10.2)
Preoperative weight loss (kg)	
≤3	1521 (70.3)
>3	642 (29.7)
Performance status	
0	782 (36.2)
1	1283 (59.3)
2	98 (4.5)
Comorbidity	
None	1756 (81.2)
At least 1	407 (18.8)
Complications	
No	1685 (77.9)
Yes	478 (22.1)
Anemia	
No	1578 (73.0)
Yes	585 (27.0)
Perioperative transfusion (units)	
0	1763 (81.5)
1–4	253 (11.7)
>4	147 (6.8)
TNM stage	
I	451 (20.9)
II	677 (31.3)
III	1035 (47.9)
Adjuvant chemotherapy	
No	1031 (47.7)
Yes	1132 (52.3)

LVI, lymphatic vessel infiltration; PNI, prognostic nutritional index; TNM, tumor‐node‐metastasis staging.

In stage III GC, the correlation of preoperative anemia status with clinicopathologic characteristics is shown in Table 2. The presence of preoperative anemia was associated with age ≥60 years (*P *=* *0.002), male patients (*P *=* *0.026), larger tumor size (*P *<* *0.001), elevated PNI (*P *<* *0.001), more preoperative weight loss (*P *=* *0.007), poor performance status (*P *=* *0.006), incidence of postoperative complications (*P *<* *0.001), and more perioperative transfusions (*P *<* *0.001).

**Table 2:** Correlation of preoperative anemia status with clinicopathologic characteristics in stage III gastric cancer patients

Characteristic	Anemic (*n* = 314)	Nonanemic (*n* = 721)	*P*‐value
Age (years)			0.002
<60	150	419	
≥60	164	302	
Sex			0.026
Male	123	231	
Female	191	490	
Tumor size (cm)			<0.001
<5	81	320	
≥5	233	401	
Tumor location			0.142
Upper third	105	281	
Middle third	99	190	
Lower third	110	250	
Grade			0.122
G1/G2	48	85	
G3/G4	266	636	
Lauren histotype			0.852
Intestinal	51	134	
Diffuse/Mixed	152	387	
LVI			0.593
Absent	201	443	
Present	22	46	
PNI			<0.001
0	254	668	
1	60	53	
Preoperative weight loss (kg)			0.007
≤3	182	502	
>3	132	219	
Performance status			0.006
0	90	248	
1	198	445	
2	26	28	
Comorbidity			0.889
None	225	514	
At least 1	89	207	
Complications			<0.001
No	178	526	
Yes	136	195	
Perioperative transfusion (units)			<0.001
0	159	667	
1–4	88	44	
>4	67	10	
pT stage			0.099
pT2	4	27	
pT3	114	252	
pT4	196	442	
pN stage			0.291
pN0/pN1	58	114	
pN2/pN3	256	607	
Dissected lymph nodes			0.734
≤21	132	322	
22–29	79	162	
>29	103	237	
TNM stage			0.930
IIIa	103	237	
IIIb	100	237	
IIIc	111	247	
Adjuvant chemotherapy			0.385
No	103	210	
Yes	211	511	

LVI, lymphatic vessel infiltration; PNI, prognostic nutritional index; TNM, tumor‐node‐metastasis staging.

**Figure 2:**
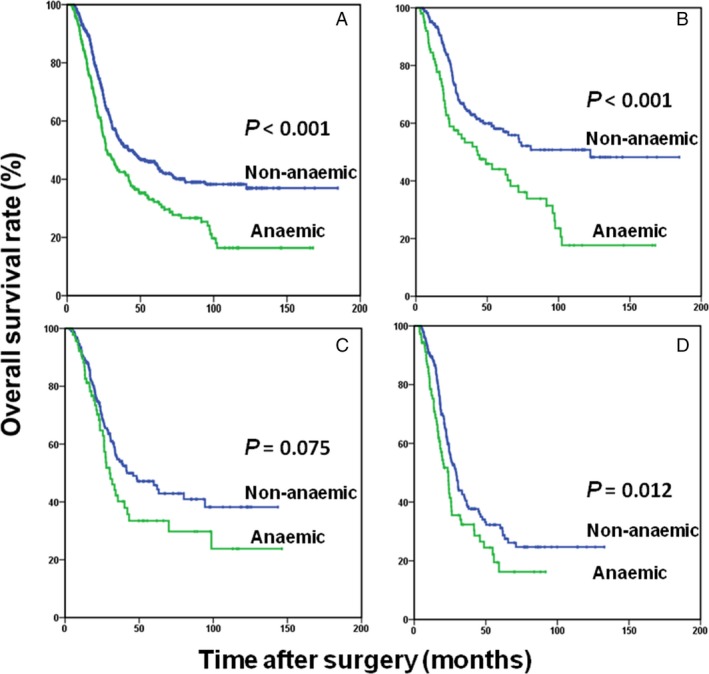
Overall survival based on preoperative anemia status in patients with stage III (A), stage IIIa (B), stage IIIb (C), and stage IIIc (D) gastric cancer.

The results of the Cox proportional hazards regression model used to identify prognostic risk factors for OS are shown in Table 3. In patients with stage III GC, 10 prognostic risk factors were identified in the univariate analysis, including age, tumor size, tumor location, LVI, PNI, preoperative weight loss, complications, anemia, perioperative transfusion, and TNM stage (All *P *<* *0.05). Multivariate analysis revealed that preoperative anemia (HR, 1.771; 95% CI, 1.040–3.015; *P *=* *0.035) was an independent predictor for OS, along with age (HR, 1.522; 95% CI, 1.133–2.045; *P *=* *0.005), tumor location (HR, 0.589; 95% CI, 0.430–0.807; *P *=* *0.001), preoperative weight loss (HR, 1.570; 95% CI, 1.162–2.122; *P *=* *0.003), and 7th TNM stage (HR, 1.518; 95% CI, 1.236–1.864; *P *<* *0.001). In a stage‐stratified analysis, it was still independently associated with OS in TNM stages IIIa through IIIc (*P *<* *0.001, *P *=* *0.075, and *P *=* *0.012, respectively; Fig. 2), though the association was marginal in stage IIIb. By the Cox proportional hazards regression models, the results were further validated in stage IIIa (HR, 1.469; 95% CI, 1.010–2.138; *P *=* *0.044), stage IIIb (HR, 1.364; 95% CI, 0.993–1.875; *P *=* *0.056), and stage IIIc patients (HR, 1.530; 95% CI, 1.123–2.084; *P *=* *0.007), respectively.

**Table 3:** Prognostic factors for overall survival in 1035 patients with stage III gastric cancer undergoing curative resection

	Univariate analysis		Multivariate analysis	
	HR (95% CI)	*P*‐value	HR (95% CI)	*P*‐value
Age (years)		<0.001		0.005
<60	1.00		1.00	
≥60	1.648 (1.376, 1.975)		1.522 (1.133, 2.045)	
Sex		0.472		
Female	1.00			
Male	0.932 (0.770, 1.129)			
Tumor size (cm)		0.001		0.671
<5	1.00		1.00	
≥5	1.397 (1.156, 1.689)		0.938 (0.696, 1.262)	
Tumor location		<0.001		0.001
Upper/Middle third	1.00		1.00	
Lower third	0.673 (0.553, 0.819)		0.589 (0.430, 0.807)	
Grade		0.715		
G1/G2	1.00			
G3/G4	1.052 (0.801, 1.382)			
Lauren histotype		0.491		
Intestinal	1.00			
Diffuse/Mixed	0.863 (0.566, 1.314)			
LVI		0.016		0.560
Absent	1.00		1.00	
Present	1.527 (1.083, 2.153)		1.153 (0.715, 1.861)	
PNI		<0.001		0.169
0	1.00		1.00	
1	1.728 (1.278, 2.336)		0.668 (0.376, 1.187)	
Preoperative weight loss (kg)		<0.001		0.003
≤3	1.00		1.00	
>3	1.564 (1.264, 1.935)		1.570 (1.162, 2.122)	
Performance status		0.935		
0	1.00			
1/2	0.992 (0.812, 1.211)			
Comorbidity		0.306		
None	1.00			
At least 1	1.203 (0.844, 1.714)			
Complications		0.005		0.131
No	1.00		1.00	
Yes	1.575 (1.145, 2.166)		0.767 (0.543, 1.083)	
Anemia		<0.001		0.035
No	1.00		1.00	
Yes	1.563 (1.293, 1.890)		1.771 (1.040, 3.015)	
Perioperative transfusion (units)		0.001		0.440
0	1.00		1.00	
1–4	1.412 (1.043, 1.912)	0.026	0.856 (0.410, 1.785)	0.678
>4	1.669 (1.205, 2.312)	0.002	0.563 (0.233, 1.358)	0.201
TNM stage		<0.001		<0.001
IIIa/IIIb	1.00		1.00	
IIIc	1.733 (1.441, 2.086)		1.518 (1.236, 1.864)	
Adjuvant chemotherapy		0.089		
No	1.00			
Yes	0.825 (0.661, 1.030)			

LVI, lymphatic vessel infiltration; PNI, prognostic nutritional index; TNM, tumor‐node‐metastasis staging.

**Figure 3:**
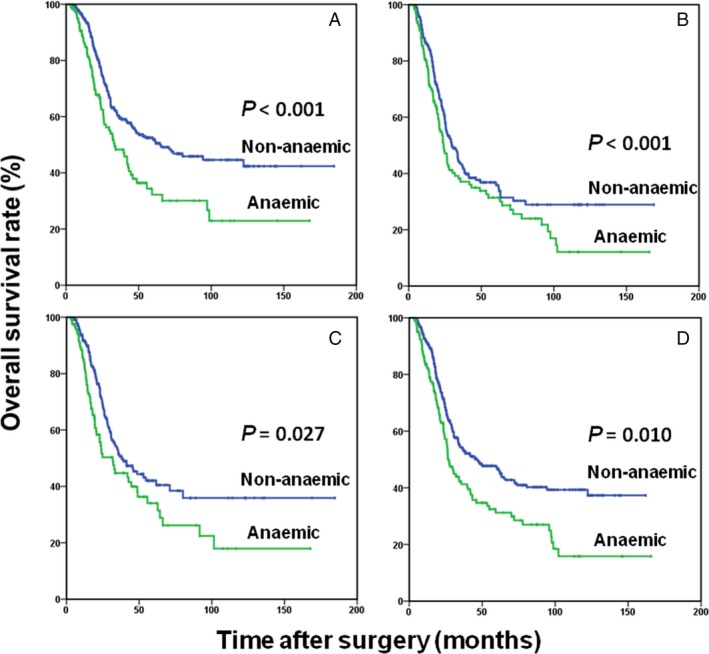
Overall survival based on the preoperative anemia status in patients age <60 years (A), age ≥60 years (B), female patients (C), and male patients (D).

Furthermore, when stratified by age and sex in TNM stage III patients, the prognostic significance of preoperative anemia was maintained in age ≥60 years patients (HR, 1.354; 95% CI, 0.989–1.855; *P *=* *0.059), age <60 years patients (HR, 1.460; 95% CI, 1.042–2.045; *P *=* *0.028), female patients (HR, 1.499; 95% CI, 1.002–2.243; *P *=* *0.049), and male patients (HR, 1.516; 95% CI, 1.144–2.010; *P *=* *0.004), respectively (Fig. 3). Of note, preoperative mild anemia had similar prognostic value in stage III GC (HR, 1.954; 95% CI, 1.103–3.460; *P *=* *0.022), even in a stage‐stratified analysis.

Finally, we performed the propensity score matching to eliminate bias to further verify our conclusions in overall patients. By the Pearson's chi‐squared test, the covariates for propensity score matching were age, sex, tumor size, PNI, preoperative weight loss, performance status, and TNM stage. After 1:1 propensity score matching, the covariates were balanced and 1150 patients were enrolled in our analysis. The Cox proportional hazards regression models were used to further validate our conclusions in overall patients (HR, 1.129; 95% CI, 0.869–1.466; *P *=* *0.363), stage I patients (HR, 1.051; 95% CI, 0.458–2.409; *P *=* *0.907), stage II patients (HR, 1.070; 95% CI, 0.837–1.369; *P *=* *0.588), and stage III patients (HR, 1.469; 95% CI, 1.125–1.917; *P *=* *0.005), respectively.

## Discussion

Anemia is a common condition in cancer patients 22, 23. In addition to the negative effect on the quality of life, the presence of pretreatment anemia has been found to be an unfavorable prognostic factor in many types of malignancies 24, 25, 26. In our study, we explored the potential prognostic value of preoperative anemia in a large cohort of Chinese patients with GC. The prevalence of anemia in patients with GC undergoing curative resection was 27.0%. We found that preoperative anemia, even if mild, was an independent prognostic risk factor for TNM stage III GC but not stage I or II.

Many studies supported that preoperative anemia was associated with poor prognosis in cancer patients. However, the underlying mechanisms remain unclear. Over the past decades, several hypotheses have been proposed to clarify this relationship. First, growing evidence has indicated that low hemoglobin levels induce poor tumor oxygenation 27. Furthermore, a hypoxic microenvironment plays an important role in the malignant phenotype of the tumor, promoting malignant progression, loco‐regional spread and distant metastasis. Accumulating evidence has indicated that intratumoral hypoxia is a key factor in the activation of hypoxia‐inducible factor‐1, which can lead to the acceleration of tumor metastasis 28, 29. Second, preoperative anemia can influence the effects of postoperative adjuvant treatment. Numerous studies have revealed that hypoxic cancer cells are likely to be resistant to chemotherapy and radiotherapy through a series of mechanisms that include proteomic and genomic changes. Furthermore, hypoxic stress protein and missed apoptotic potential can also induce the resistance of tumor cells to chemotherapy drugs 30, 31.

In fact, our findings were supported by other studies, which have also shown an association between preoperative anemia and clinical outcome. In a series of 352 patients with papillary renal cell carcinoma who underwent complete resection, Huang et al. found that preoperative anemia was independently associated with an increased risk of recurrence and mortality 32. A recent study evaluated the outcome of 367 patients with soft tissue sarcoma and found that low preoperative hemoglobin levels were significantly associated with decreased cancer‐specific survival and OS 33. Studies from our center have reported that preoperative anemia was an independent predictor of outcome in breast cancer 34. It should be noted that one similar sized study from Korea showed that preoperative anemia was an independent prognostic factor for TNM stage I‐II GC but not stage III 15. Considering regional differences, different cut‐off levels for anemia, and treatment advancements in the past decade, we further validated the prognostic value of preoperative anemia in a large cohort of Chinese GC patients.

Furthermore, we found the presence of preoperative anemia was associated with a larger tumor size. The findings were consistent with the previous studies, which have shown that preoperative anemia was associated with features of aggressive tumor biology. A larger tumor was easier to lead to tumor‐associated bleeding, abnormal catabolism and malnutrition, which contributed to a change in body composition and diminished function 35, 36. However, the potential mechanisms underlying the observed associations remain unclear. In addition, preoperative anemia was significantly associated with postoperative complications and several nutritional‐based indices, including the PNI, preoperative weight loss and performance status. In fact, our conclusions are supported by other studies 37. Data from the European Cancer Anaemia Survey highlighted the link between low hemoglobin levels and poor performance status 35. Furthermore, a study from Zhang et al. also revealed that preoperative anemia was significantly correlated with preoperative nutritional status 34. In fact, accumulating evidence has indicated that preoperative malnutrition induces poor clinical outcomes 38. Therefore, our study may add to the evidence that preoperative anemia can provide additional prognostic information in GC.

In daily clinical practice, preoperative hemoglobin levels may be helpful to tailor treatment to each individual situation. With accurate clinical prediction, nonanemic patients can avoid the potential toxicity of further treatment. However, anemic patients with stage III GC need additional systemic adjuvant therapy and closer follow‐up schedules. Of note, it is still unclear whether preoperative anemia treatment can alter the poor prognosis. Though blood transfusions are widely used for anemic patients, emerging evidence indicates that it is associated with poor clinical outcomes 39, 40. However, in this study, perioperative transfusion did not significantly influence OS in the multivariate analysis. Considering the low transfusion rate in this analysis, we should consider the conclusion with caution. Indeed, there is a body of evidence indicating that blood transfusion contributes to increased risk of treatment‐related adverse events and postoperative complications 41, which provides the rationale for exploration of better treatment options. In addition, the efficacy of intravenous iron and recombinant human erythropoietin in anemic patients with GC is also worthy of further study 42, 43. Therefore, large‐scale prospective studies are needed to validate whether early targeted intervention is beneficial to anemic patients undergoing curative resection for GC.

First, this study was limited by its single‐institution nature; however, with a large cohort of patients, our study provides a valid basis for determining the prognostic value of preoperative anemia in GC. Second, postoperative treatment heterogeneity was inevitable due to the retrospective design, which might affect our results. Third, though OS is considered the gold standard end‐point in the aspect of cancer prognosis study, we lack disease‐specific survival and relapse‐free survival data. Our study may have been strengthened by the use of other survival measures. Fourth, subgroup‐derived evidence is not strong enough to validate some conclusions, which need to be strengthened in prospective randomized controlled studies.

## Conclusions

Preoperative anemia, even when mild, is independently associated with poor outcomes in patients with TNM stage III GC. Prospective studies are needed to determine the potential impact of early targeted intervention in anemic patients undergoing curative resection for GC.

## Conflict of Interest

The authors have no conflicts of interest in this paper.

## Supporting information


**Figure S1.** Overall survival based on preoperative anemia status in patients with stage I (A) and stage II (B) gastric cancer.

**Table S1**. STROBE statement—checklist of items that should be included in the study.
