# Supplementary figures and images for: Impact of preoperative anemia on outcomes in patients undergoing curative resection for gastric cancer: a single‐institution retrospective analysis of 2163 Chinese patients
Source: Cancer Med. 2018 Jan 17;7(2):360–9. doi: 10.1002/cam4.1309 (PMC5806112; doi:10.1002/cam4.1309)

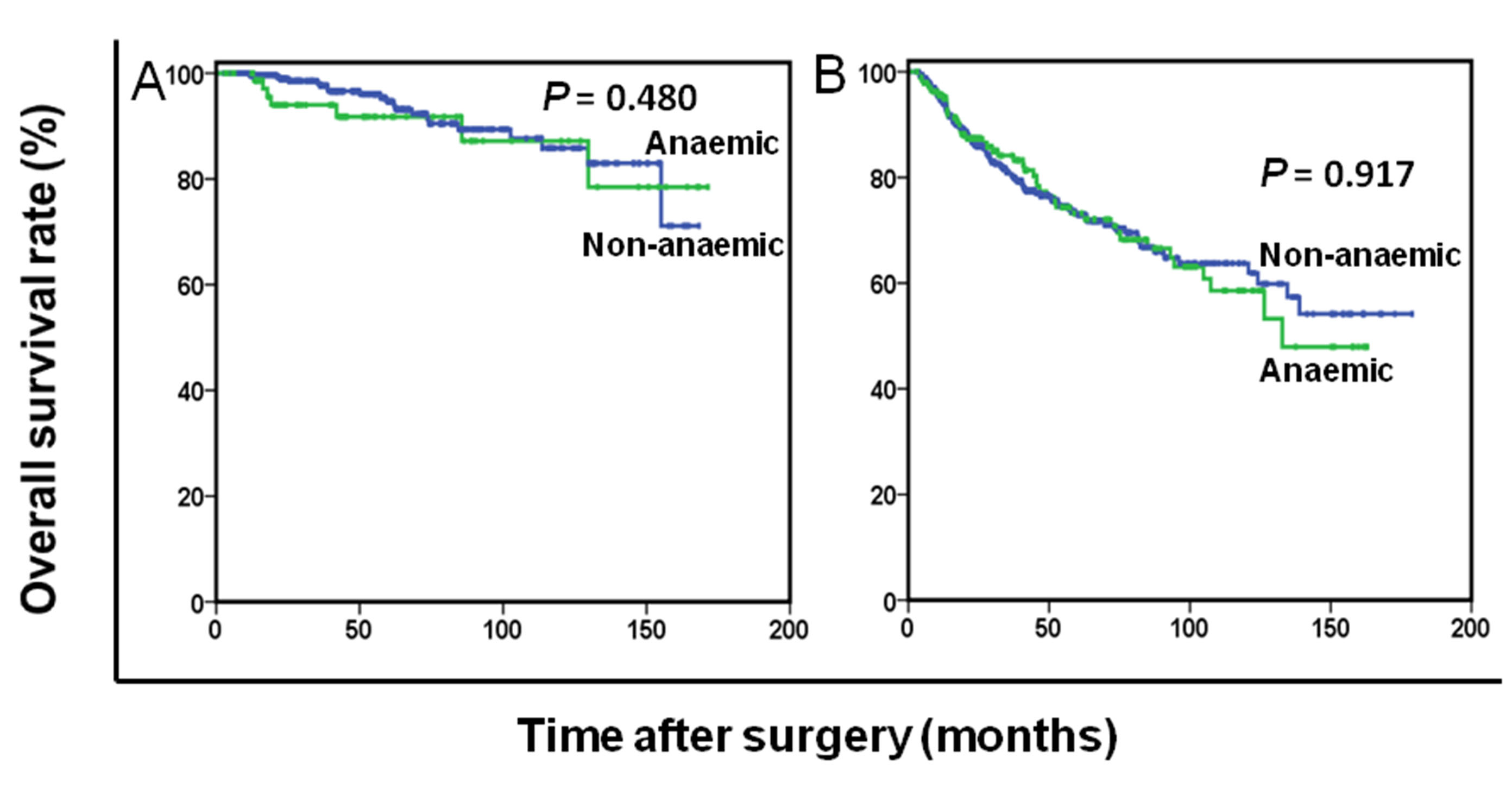

Supplement: Supplementary file 1 — Figure S1. Overall survival based on preoperative anemia status in patients with stage I (A) and stage II (B) gastric cancer. [file CAM4-7-360-s001.tif]
